# Dementia Risk of Direct Oral Anticoagulants Versus Warfarin for Atrial Fibrillation: Systematic Review and Meta-Analysis

**DOI:** 10.1016/j.jacasi.2023.07.012

**Published:** 2023-09-05

**Authors:** Khi Yung Fong, Yiong Huak Chan, Yue Wang, Colin Yeo, Barbara Helen Rosario, Gregory Y.H. Lip, Vern Hsen Tan

**Affiliations:** a Yong Loo Lin School of Medicine, National University of Singapore, Singapore; b Biostatistics Unit, Yong Loo Lin School of Medicine, National University of Singapore, Singapore; c Department of Cardiology, Changi General Hospital, Singapore; d Department of Geriatric Medicine, Changi General Hospital, Singapore; e Liverpool Centre for Cardiovascular Science at University of Liverpool, Liverpool John Moores University and Liverpool Heart & Chest Hospital, Liverpool, United Kingdom; f Department of Clinical Medicine, Aalborg University, Aalborg, Denmark

**Keywords:** anticoagulants, atrial fibrillation, dementia, meta-analysis, warfarin

## Abstract

**Background:**

Direct-acting oral anticoagulants (DOACs) have demonstrated superior efficacy in preventing stroke and death compared with warfarin in patients with atrial fibrillation (AF), but their influence on dementia risk remains unclear.

**Objectives:**

The purpose of this study was to evaluate the relative risks of dementia in DOAC vs warfarin in patients with AF.

**Methods:**

An electronic literature search was conducted to retrieve studies reporting comparisons of dementia incidence between patients treated with DOACs and warfarin for AF. HRs and 95% CI were pooled in a random-effects meta-analysis. Meta-regression was performed to identify prognostic baseline variables. Network meta-analysis was performed to determine dementia risk between individual DOACs and warfarin.

**Results:**

Ten studies (n = 342,624) were retrieved. DOAC was associated with a significantly lower risk of developing dementia compared with warfarin (HR: 0.88; 95% CI: 0.80-0.98; *P* = 0.017; I^2^ = 75%); significance was also seen in Asian patients (HR: 0.81; 95% CI: 0.68-0.86) but not non-Asian patients. Subgroup analyses of propensity score–matched studies and patients aged 65-75 years showed similar significance, but not for patients aged ≥75 years. Meta-regression found that a lower mean age corresponded to significantly greater favoring of DOAC over warfarin. Network meta-analysis found significant reductions in dementia risk over warfarin for rivaroxaban (HR: 0.854; 95% CI: 0.763-0.955), apixaban (HR: 0.881; 95% CI: 0.778-0.997), and dabigatran (HR: 0.871; 95% CI: 0.770-0.987); the highest-ranked treatment based on P scores was edoxaban.

**Conclusions:**

The use of DOAC in AF significantly reduces dementia risk compared with warfarin, particularly in Asian patients. The possible reversal of this effect with increasing age merits further randomized trials with long-term follow-up. (Dementia Risk of Direct Oral Anticoagulants Versus Warfarin for Atrial Fibrillation: A Systematic Review and Meta-Analysis; CRD42022365634)

Atrial fibrillation (AF) is a prevalent condition among older people, with a lifetime risk ranging from 1 in 3 to 1 in 5,^1^ and is associated with a 5-fold risk of stroke.^2^ Increased risks of cognitive impairment and dementia have also been demonstrated.^3^ Although stroke is a known contributory factor to dementia, particularly vascular dementia,^4^ the association between AF and Alzheimer’s dementia has been suggested to be independent of the occurrence of stroke.^5^ Yet, it remains unknown whether AF is a direct causal factor for cognitive decline, or is simply a marker of global vascular disease burden. This has led to increasing interest in the “heart-brain axis,” with a call for larger longitudinal studies to tease apart the multifactorial relationships between AF and cognitive dysfunction.^6^

Oral anticoagulants (OACs) such as warfarin and direct-acting oral anticoagulants (DOACs) are central for stroke prevention in patients with AF.^2^^,^^7^ In several pivotal trials, DOACs have demonstrated similar or superior efficacy in preventing stroke and death, as well as lower rates of hemorrhage compared with warfarin.^8^, ^9^, ^10^ Maintaining a proper international normalized ratio (INR) is also crucial when using warfarin because lower time in therapeutic range (TTR) has been associated with higher risk of dementia.^11^ Nonetheless, studies comparing the risk of dementia in DOAC vs warfarin have yielded conflicting results, with some showing reduced risk with DOAC and others finding no significant difference.^12^ Crucially, previous studies with small sample sizes were not sufficiently powered to detect any differences in dementia risk, which can only be detected in large cohorts.

Given the uncertainty in the field, this systematic review and meta-analysis aims to compare the relative risks of dementia in patients with AF taking DOAC vs warfarin, and examine the impact of baseline demographics on these risks.

## Methods

### Literature search

This systematic review and meta-analysis was performed in line with the Preferred Reporting Items for Systematic Reviews and Meta-Analyses Guidelines and registered with PROSPERO (CRD42022365634).

An electronic literature search from inception to June 23, 2022, was conducted by 2 independent investigators (Khi Fong and Vern Tan) on PubMed, EMBASE, and Web of Science for relevant articles, using the concepts of dementia, atrial fibrillation, DOAC, and anticoagulants (Supplemental Table 1). No language restrictions were applied. Bibliographies of included studies were screened, and a search on Google Scholar using the first and last author of each included study was conducted, to ensure inclusion of all relevant studies. Retrieved abstracts and full texts were reviewed by 2 independent investigators, with conflicts being resolved via group consensus among all authors.

Prospective or retrospective studies reporting comparisons of the outcome of dementia incidence between patients treated with DOAC vs patients treated with warfarin for AF were included. Case reports, case series, reviews, and conference abstracts were excluded.

A standardized data collection template with predefined data fields including study characteristics, patient demographics and outcomes was used for data extraction by 2 independent investigators. Studies were assessed for risk of bias using the Newcastle-Ottawa Scale.

### Meta-analysis

The primary outcome in this meta-analysis was the incidence of dementia during follow-up. This outcome was analyzed in several ways. First, HRs for development of dementia and their 95% CIs were pooled in a random-effects meta-analysis. Where studies provided both raw and corrected HR estimates, the corrected HR was used. This forest plot was also stratified according to the region in which the study was conducted. Next, subgroup analysis of HR was performed for: 1) studies reporting stratified outcomes for patients ≥75 years of age and 65-75 years of age; and 2) studies that were propensity score-matched (PSM). Finally, numbers of dementia diagnoses and person-years of follow-up were pooled to determine incidence rate ratios.

Where PSM was used in the study and HRs were clearly provided for the matched group, these were preferentially used instead of HRs for the unmatched cohort.^13^ If studies did not specify summary HRs across all subgroups (eg, different age groups) and instead reported them separately, they were considered as distinct studies and pooled separately in the meta-analysis. If there was any suspected overlap in these separately reported groups due to PSM, the group with the largest number of analyzed participants was used for analysis. Random-effects Mantel-Haenszel or Inverse-Variance models were used for all analyses due to heterogeneity in definitions of dementia and study region, due to lack of specification of DOAC dosages and durations in some studies, and due to support for generalization inferences beyond the included studies. Heterogeneity was considered low, moderate, or considerable for I^2^ values <40%, 40%-75%, and >75%, respectively.^14^ Funnel plot symmetry was visually assessed for publication bias. Certainty of evidence was assessed using the Grading of Recommendations Assessment, Development and Evaluation approach.^15^

Meta-regression was performed to identify variables that had an influence on the results of the meta-analysis. Baseline variables that were reported in ≥10 included studies were used for meta-regression against the HR of respective studies. Bubble plots were generated for any significant associations. Prior to meta-regression, missing means from medians, ranges, and IQRs were derived via imputation.^16^^,^^17^

### Network meta-analysis

If ≥2 studies reported HRs of dementia risk for DOAC vs warfarin stratified by component DOACs, a network meta-analysis (NMA) was conducted. NMA allows comparison of multiple interventions to each other via pooling of indirect evidence, shedding light on treatment comparisons that have little or no head-to-head research data such as comparisons between individual DOACs. HRs were pooled in a random-effects, 2-stage Frequentist NMA, using warfarin as the common comparator. Despite overlap in the PSM warfarin cohorts for some studies, HRs were considered as separate studies due to different numbers of matched warfarin subjects to each DOAC. *I*^2^ and Cochran Q were used to test for heterogeneity and inconsistency. A network graph, league table, and forest plot were also generated for this analysis. P scores were used to numerically rank the treatment strategies in the overall cohort as well as within subgroups, with higher P scores corresponding to greater efficacy. P scores are based solely on HRs and standard errors of the frequentist NMA estimates under the normality assumption, providing an intuitive way to appraise treatments and inform medical decision-making.^18^

All analyses were performed in RStudio using R-4.1.2 and the packages “meta,” “netmeta,” and “metafor,” with *P* < 0.05 regarded to indicate statistical significance. There was no funding source for this study. This article made use of publicly available data from published studies, hence ethics approval was not required.

## Results

### Study selection

**Figure 1:**
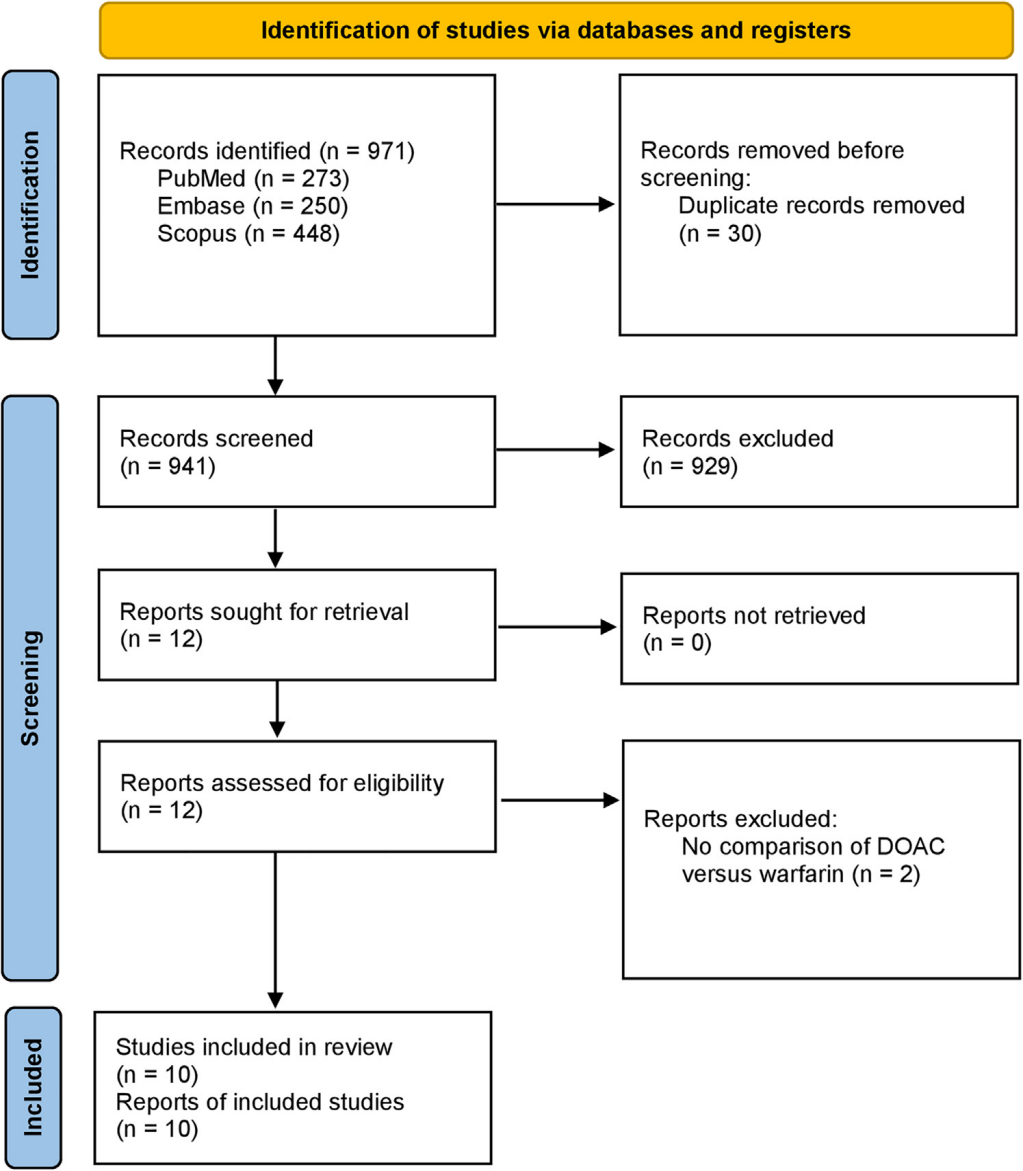
PRISMA Flowchart of Included Studies The PRISMA (Preferred Reporting Items for Systematic Reviews and Meta-Analyses) flowchart details the screening process for meta-analyses. The search strategy identifies a large number of studies, which are screened by abstract followed by full-text, and the final short-listed studies are described in this article. DOAC = direct-acting oral anticoagulants.

The search strategy yielded 971 studies. After removal of 30 duplicates, 941 studies underwent title and abstract screening. Twelve studies were identified for full-text review. Finally, 10 studies^19^, ^20^, ^21^, ^22^, ^23^, ^24^, ^25^, ^26^, ^27^, ^28^ and 342,624 patients were analyzed (Figure 1, Table 1).

**Table 1:** Characteristics of Included Studies

First Author, Year	Country	Study Type	Arm	N	Males, %	Age, y	Follow-Up Time, y	CHA_2_DS_2_-VASc Score	Heart Failure, %	DM, %	HTN, %	Previous Stroke, %	Statin Use, %
Bezabhe, 2022	Australia	PSM	DOAC	1,335	56.3	74.8 ± 9.2	3.2 ± 1.7	2.7 ± 1.3	14.3	21.7	60.5	NR	63.8
			Warfarin	1,335	56.3	75.0 ± 10.6	3.3 ± 1.4	2.7 ± 1.3	16.8	22.3	61.3	NR	64.1
Cadogan, 2021	United Kingdom	Cohort	DOAC	18,513	55.5	76 (68-83)	1.4 (0.6-2.7)	NR	20.8	24.6	67.0	18.6	67.9
			Warfarin	20,687	55.3			NR	25.3	26.8	69.2	18.3	69.9
Chen, 2018	USA	PSM	Rivaroxaban	61,641	38.7	68.1	1.4	3.3^a^	32.0^a^	30.0^a^	79.0^a^	20.0^a^	57.0^a^
			Warfarin	61,641	39.4	69.1	1.2	3.6^a^	27.0^a^	32.0^a^	75.0^a^	23.0^a^	58.0^a^
Friberg, 2017	Sweden	PSM	DOAC	7,349	57.5	71.7	0.3^a^	2.75	13.6	11.2	53.9	8.6	30.9
			Warfarin	7,349	58.0	71.3		2.70	13.6	11.4	53.0	8.3	29.9
Hsu, 2021	Taiwan	PSM	DOAC	6,034	59.5	70.3 ± 11.7	3.3	2.9 ± 1.8	35.7	38.4	80.9	34.8	32.7
			Warfarin	6,034	59.0	70.4 ± 11.6	3.1	3.0 ± 1.9	36.9	38.9	81.3	34.2	32.7
Jacobs, 2016	USA	PSM	DOAC	2,627	59.6	71.2 ± 11.8	0.8 ± 0.8	NR	30.5	29.5	76.5	10.8	NR
			Warfarin	2,627	58.4	73.5 ± 9.6	1.1 ± 0.9	NR	22.7	31.4	80.0	10.7	NR
Kim, 2021	Korea	PSM	DOAC	10,193	59.8	71 (65-77)	1.5 ± 0.8^a^	4 (3-6)	57.5	31.5	81.8	32.5	36.4
			Warfarin	10,193	59.6	72 (63-78)	1.5 ± 0.8^a^	4 (3-6)	57.7	31.7	82.4	32.7	37.3
Lee, 2021	Korea	Cohort	DOAC	46,898	56.5	72.7 ± 9.9	1.3	4.1 ± 1.7	45.6	27.1	85.8	25.0	NR
			Warfarin	25,948	59.5	70.1 ± 11.2		3.8 ± 1.9	40.8	25.4	81.1	27.0	NR
Mongkhon, 2020	United Kingdom	Cohort	DOAC	4,657	55.3	74.3 ± 10.3	2.2 ± 0.9	2.2 ± 1.2	9.0	2.9	3.4	10.7	58.9
			Warfarin	12,880	55.8	74.4 ± 9.7	3.8 ± 1.5	2.3 ± 1.2	9.3	2.6	3.6	9.0	58.5
Sogaard, 2019 (60-69 y)	Denmark	Cohort	DOAC	6,846	62.2	65.9 ± 2.7	3.4	2.1 ± 1.2	11.2	11.1	58.3	0 (patients with prior stroke excluded from analysis)	35.3
			Warfarin	4,332	64.4	65.9 ± 2.7	4.3	2.2 ± 1.2	16.9	14.2	60.7		37.4
Sogaard, 2019 (70-79 y)			DOAC	8,126	53.1	74.7 ± 2.9	3.1	3.1 ± 1.2	15.5	11.6	62.2		38.6
			Warfarin	5,387	56.1	74.9 ± 2.8	3.9	3.3 ± 1.3	21.1	13.6	65.0		43.3
Sogaard, 2019 (≥80 y)			DOAC	6,339	37.9	86.1 ± 4.4	2.7	3.9 ± 1.1	34.2	10.8	65.0		30.3
			Warfarin	3,653	44.5	85.1 ± 3.8	3.3	4.0 ± 1.2	15.5	11.6	69.1		34.4

HTN = hypertension; PSM = propensity score–matched study.

a Values taken from unmatched cohort.

### Study characteristics

There were 6 PSM^19^^,^^21^, ^22^, ^23^, ^24^, ^25^ and 4 unmatched cohort^20^^,^^26^, ^27^, ^28^ studies. One cohort study, Søgaard et al,^28^ stratified their population into 3 age groups (60-69, 70-79, and ≥80 years) without summary HR outcomes for the whole cohort. One PSM study, Chen et al^21^ performed separate instances of propensity matching between 1 warfarin cohort and each of 3 DOAC cohorts (rivaroxaban, apixaban, and dabigatran); due to the duplication in patients from the matched warfarin arm that would occur if all 3 matched cohorts were pooled, the cohort with the largest number of patients (rivaroxaban vs warfarin) was selected for subsequent analysis. Five studies provided HRs for patients ≥75 years of age, of which one, Friberg et al^22^ provided separate HRs for patients 75-85 years and ≥85 years; 3 studies provided HRs for patients 65-75 years of age.

The mean age of participants ranged from 70.4-75.7 years, with a slight male predominance (50.6%). Follow-up time ranged from 0.3-3.7 years. The presence of heart failure or diabetes mellitus at baseline was <50%, but the majority of patients had hypertension. The presence of previous stroke ranged from 0%-27%. The dementia endpoint in all studies was determined by reviewing the incidence of new disease codes for a dementia diagnosis after anticoagulant initiation from the respective patient databases. Risk of bias was generally low (Supplemental Table 2).

### Statistical analysis

**Figure 2:**
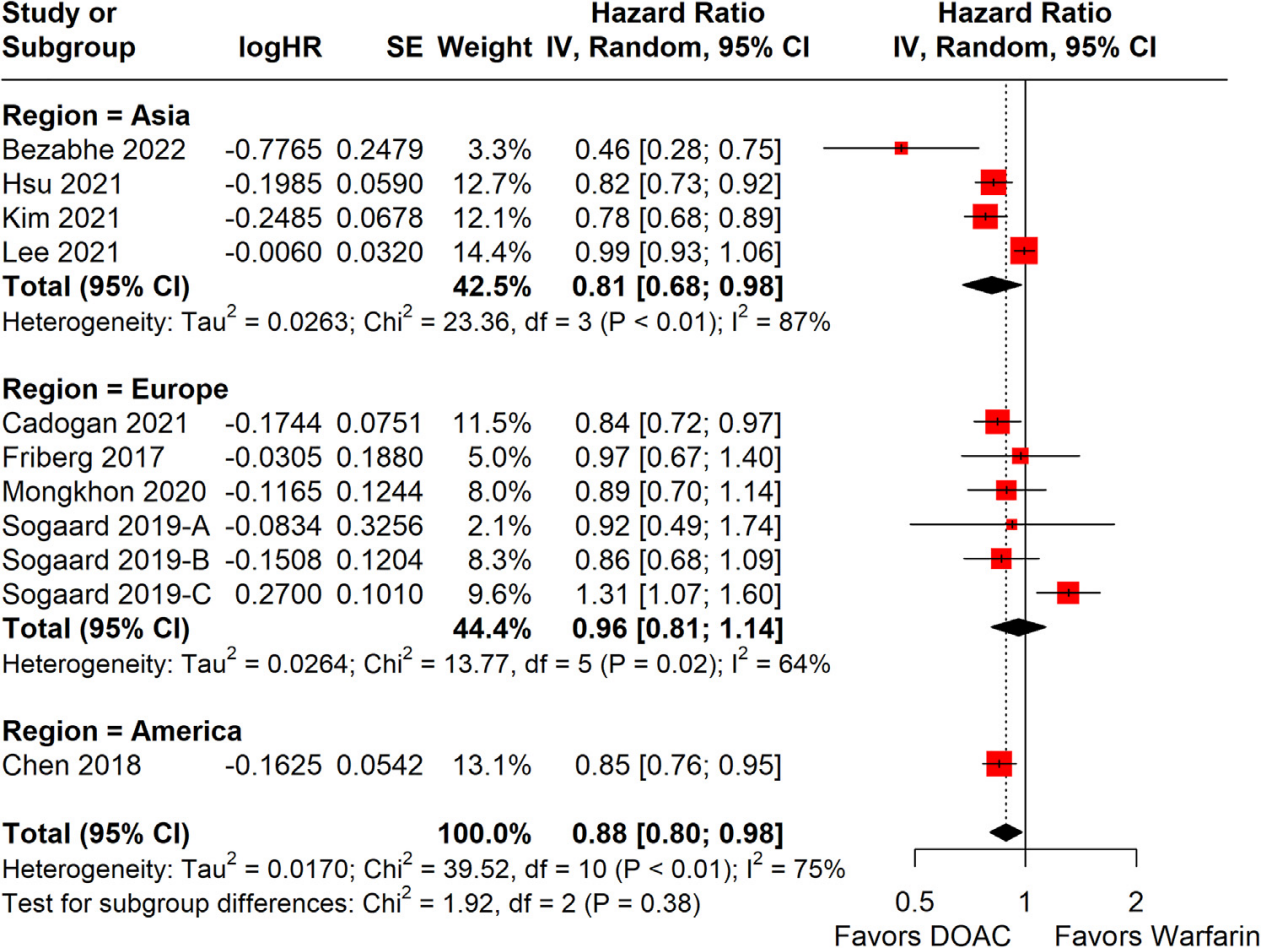
Random-Effects Meta-Analysis of HR for Dementia Development HRs for development of dementia across study follow-up are shown in this forest plot, stratified by region. Summary hazard regions for each region, and across all studies, are bolded. Sogaard 2019-A, -B, and -C refer to age groups of 60-69 years, 70-79 years, and ≥80 years, respectively. IV = inverse-variance; SE = standard error of treatment effect; TE = treatment effect; other abbreviations as in Figure 1.

**Figure 3:**
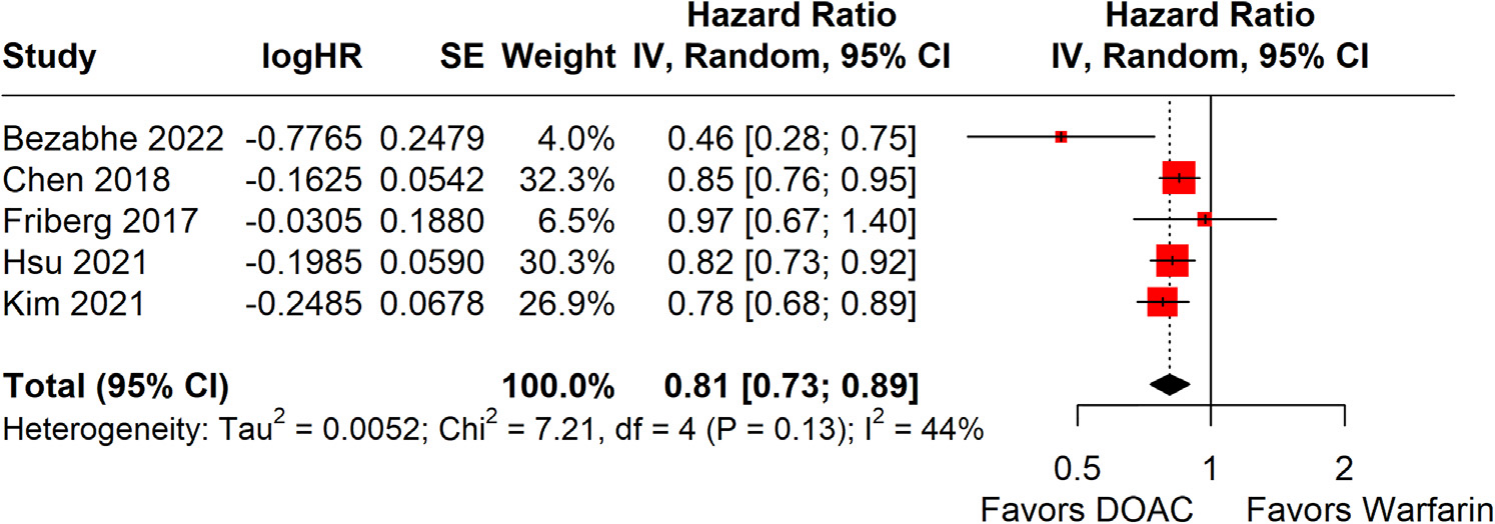
Subgroup Analysis of Dementia Development in Propensity Score–Matched Studies HRs for development of dementia across study follow-up are shown in this forest plot, which only includes studies using propensity score–matching. Abbreviations as in Figures 1 and 2.

Among 9 studies,^19^, ^20^, ^21^, ^22^, ^23^^,^^25^, ^26^, ^27^, ^28^ one of which had 3 separate arms stratified by age, DOAC was associated with a significantly lower risk of developing dementia compared with warfarin (HR: 0.88; 95% CI: 0.80-0.98: *P* = 0.017; *I*^2^ = 75%) (Figure 2). When stratified by region, a benefit for DOAC was seen in Asian patients (4 studies;^19^^,^^23^^,^^25^^,^^26^ HR: 0.81; 95% CI: 0.68-0.86) but not European patients (4 studies;^20^^,^^22^^,^^27^^,^^28^ HR: 0.96; 95% CI: 0.81-1.14). Only 1 study^21^ was available for the American region. Similar significance was seen in the subgroups of PSM studies (HR: 0.81; 95% CI: 0.73-0.89; *P* < 0.001; *I*^2^ = 44%) (Figure 3) and patients aged 65-75 years old (HR: 0.80; 95% CI: 0.70-0.91; *P* < 0.001; *I*^2^ = 0%) (Supplemental Figure 1) but not for the subgroup of patients aged ≥75 years old, which had a trend favoring DOAC that did not reach statistical significance (HR: 0.94; 95% CI: 0.87-1.02; *P* = 0.140; *I*^2^ = 23%) (Supplemental Figure 2). Among the 9 studies, the incidence rate of dementia was significantly lower in DOAC compared with warfarin (incidence rate ratio: 0.87; 95% CI: 0.76-1.00; *P* = 0.045; *I*^2^ = 87%) (Supplemental Figure 3). Funnel plots were visually symmetrical and not suggestive of publication bias (Supplemental Figures 4 to 8). Level of evidence was rated as moderate via the Grading of Recommendations Assessment, Development and Evaluation approach (Supplemental Table 3).

For meta-regression, baseline demographics variables, which were reported in 10 or more of the studies, pooled in the analysis of HR were as follows: year of publication, mean age, mean follow-up time, percentage of males in the study, CHA_2_DS_2_-VASc score, heart failure, diabetes mellitus, hypertension, previous stroke, and statin use. A significant association between mean age and HR for dementia was found, with a lower mean age corresponding to a greater favoring of DOAC over warfarin (β = 0.023; 95% CI: 0.002-0.043; *P* = 0.03; *I*^2^ = 70%) (Table 2, Supplemental Figure 9). No other significant associations were found.

**Table 2:** Meta-Regression of Baseline Characteristics Against HR of DOAC Vs Warfarin

	Studies	**β**	95% CI	*I*^2^	*R*^2^	*P* Value
Publication year	11	−0.057	−0.138 to 0.024	77	0	0.166
Mean age, y	11	0.023	0.002-0.043	70	24	**0.030**
Follow-up time	10	−0.017	−0.123 to 0.090	76	0	0.762
Male %	11	0.003	−0.018 to 0.024	77	0	0.794
CHA_2_DS_2_-VASc score	10	0.097	−0.034 to 0.228	72	15	0.147
Heart failure	11	0	−0.007 to 0.008	77	0	0.899
HTN	11	0	−0.005 to 0.005	77	0	0.914
DM	11	−0.007	−0.015 to 0.002	71	19	0.142
Previous stroke	10	−0.007	−0.014 to 0.000	68	18	0.064
Statin use	10	−0.005	−0.014 to 0.003	69	0	0.184

Bold indicate significance (*P* < 0.05).

DM = diabetes mellitus; DOAC = direct-acting oral anticoagulant; HTN = hypertension.

**Figure 4:**
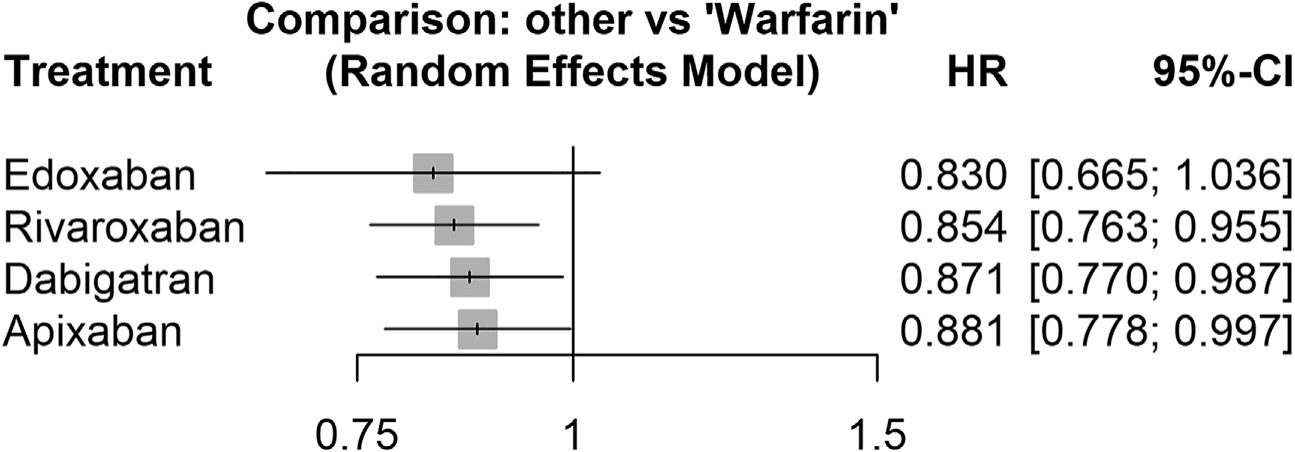
Network Meta-Analysis of Dementia Risk Across Individual DOACs and Warfarin HRs for dementia risk of each DOAC vs warfarin in all studies reporting this stratification were pooled in a random-effects network analysis. Abbreviations as in Figure 1.

In the NMA, 3 studies^21^^,^^25^^,^^26^ provided stratified data for individual DOAC agents vs warfarin. Rivaroxaban, dabigatran, and apixaban demonstrated significant reduction in dementia risk over warfarin, with the upper bound of all 3 CIs <1 (Figure 4). The reduction in dementia risk for edoxaban was not significant (pooled HR: 0.830; 95% CI: 0.665-1.036), but this was likely due to its use in only 1 study. Moderate heterogeneity was observed (*I*^2^ = 62.4%; *Q* = 23.9; *P* = 0.004). P scores ranked edoxaban as the therapy with the numerically highest reduction in dementia risk compared with warfarin, followed by rivaroxaban, dabigatran, and apixaban, but no significant differences between DOACs were seen in the league table (Table 3). The network plot is shown in Supplemental Figure 10.

**Table 3:** League Table for Network Meta-Analysis of Dementia Risk

**Edoxaban (*P* = 0.715)**				
0.972 (0.758-1.247)	**Rivaroxaban (*P* = 0.668)**			
0.953 (0.739-1.228)	0.980 (0.840-1.143)	**Dabigatran (*P* = 0.572)**		
0.942 (0.731-1.215)	0.969 (0.836-1.123)	0.989 (0.839-1.167)	**Apixaban (*P* = 0.523)**	
0.830 (0.665-1.036)	*0.854 (0.763-0.955)*	*0.871 (0.770-0.987)*	*0.881 (0.778-0.997)*	**Warfarin (*P* = 0.022)**

The table should be read from left to right. HRs and 95% CIs for development of dementia for each comparison are in the cell in common between the column-defining and row-defining treatment. A HR of <1 favors column-defining treatment (lower risk of dementia). Significant comparisons are highlighted in *italics*. P scores for each treatment are provided in the same cell.

## Discussion

This meta-analysis presents a comprehensive summary of the existing literature, demonstrating that DOAC use in AF is indeed associated with a reduced risk of dementia compared with warfarin (Central Illustration). This effect was seen even when data was restricted to PSMs only, and on NMA of individual DOACs vs warfarin. Significance was seen for the subset of Asian patients but not non-Asian patients. Included studies had large cohorts and were sufficiently powered to detect differences in dementia risk.

**Central Illustration:**
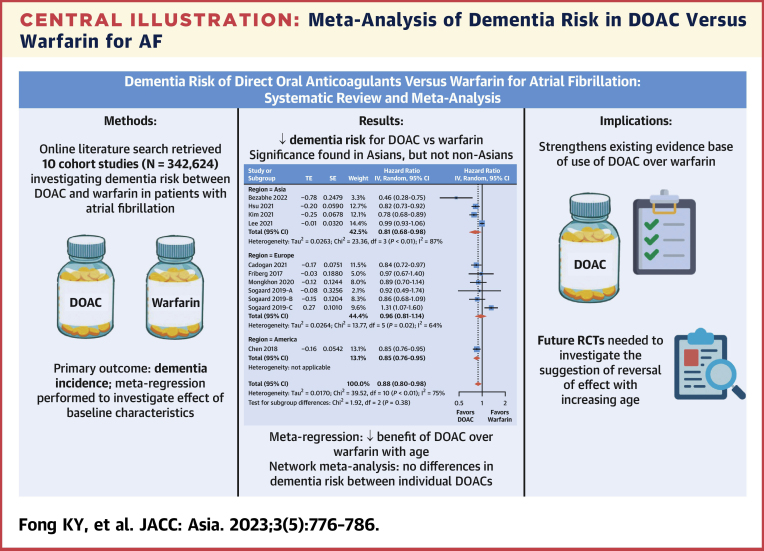
Meta-Analysis of Dementia Risk in DOAC Versus Warfarin for AF This meta-analysis of 10 large cohort studies illustrates yet another clinical endpoint in which DOAC may be favored over warfarin in patients with AF. AF = atrial fibrillation; DOAC = direct-acting oral anticoagulant; IV = inverse-variance; RCT = randomized controlled trial; SE = standard error of treatment effect; TE = treatment effect.

Previous meta-analyses demonstrated a similar reduction in dementia risk with DOAC use as opposed to warfarin.^29^^,^^30^ However, they were limited by the inclusion of data from the pivotal randomized trials comparing DOAC vs warfarin. Information on dementia diagnoses were only posted on the corresponding trial websites of the National Library of Medicine and not analyzed directly in the final manuscripts, the numbers of dementia cases were too low due to studies being underpowered to detect differences in this outcome, and time-to-event analysis in the form of HR was not provided. These trials were excluded from the present analysis to reduce the risk of bias and heterogeneity.

Several mechanisms linking AF to an increased risk of dementia have been proposed. Stroke has been proposed as a significant contributory factor to the pathogenesis of dementia, with studies showing that new-onset dementia and cognitive impairment are the sequelae of stroke.^31^^,^^32^ Nonetheless, other evidence supports a stroke-independent risk of dementia.^33^^,^^34^ Explanations for this include silent stroke from microembolization, subclinical cerebral hypoperfusion,^35^ and enhancement of inflammatory response by AF.^36^

These mechanisms, although not directly linked to the occurrence of overt stroke, are closely related in pathophysiology and may similarly be prevented with OAC.^36^ The lower risk of intracranial bleeding and stroke observed with DOAC compared with warfarin theoretically translates to lower rates of microembolization and cerebral hypoperfusion. Moreover, given the marked influence of TTR and labile INR on dementia and intracranial bleed risk in patients on warfarin,^11^ the use of DOAC may provide better control of coagulation profile and prevent the aforementioned pathways, which may lead to dementia.

From a dietary standpoint, patients taking a DOAC would not require limitation of vitamin K intake, unlike those on warfarin. Low dietary intake or low blood levels of vitamin K have been associated with cognitive decline and Alzheimer’s disease.^37^ Furthermore, green leafy vegetables—a source of vitamin K—are also high in vitamin B_12_ and folate, which are associated with lower incidences of cognitive decline and dementia.^38^ Hence, the benefits of DOAC over warfarin may not be solely related to the effect of DOAC alone.

Meta-regression found that higher age was associated with lower dementia risk with warfarin compared with DOAC. These findings are at odds with studies of nondementia outcomes in the literature, with DOAC demonstrating continued benefit over warfarin even in patients of advanced age, with a cohort study even deeming DOAC of greatest benefit in the very elderly.^39^ It is uncertain whether confounders, such as the nature of AF (paroxysmal, persistent, or permanent), type and dose of DOAC, and TTR of warfarin users, may have impacted these results. These baseline variables were infrequently reported and insufficient studies reporting this outcome were available for meta-regression. Pharmacokinetic differences between OACs—DOACs with predominantly renal clearance and warfarin with predominantly hepatic clearance—could have led to altered effects of DOACs in older patients because glomerular filtration rate decreases and chronic kidney disease increases with age.^40^ Dose reductions in DOACs for patients of more advanced age may have led to differing effects from younger patients. Altered pharmacokinetics may have led to suboptimal dosing of DOACs and a resulting worsening of dementia risk compared with warfarin. Nonetheless, investigations into DOAC use in renal impairment have yielded mixed results.^41^, ^42^, ^43^ The prevalence of renal disease ranged from 1.7%-30.4% among included studies, although insufficient studies were present for meta-regression. Thus, further studies are required to investigate this correlation.

A significantly lower risk of dementia was seen in DOACs compared with warfarin for the subgroup of Asian patients, but not European patients. A similar discordance has been previously reported for the setting of venous thromboembolism prophylaxis.^44^ Physiologically, Asian patients are likely to be sensitive responders to vitamin K antagonism via the CYP2C9 and VKORC1 genotypes, placing them at a higher risk of bleeding events.^45^^,^^46^ These bleeding events may either occur intracranially and contribute to dementia development or occur extracranially and result in a dose reduction and subtherapeutic INR that predisposes to thrombotic events. Moreover, due to the generally lower body weight in Asians compared with non-Asians, DOACs may exert supratherapeutic effects in Asians at standard doses. A meta-analysis found that low-dose DOAC was in fact equivalent to standard-dose DOAC in terms of embolic and bleeding endpoints.^47^ Conversely, in the heavier non-Asian population, subtherapeutic dosing of DOACs may lead to greater thrombotic events, compared with warfarin where INR is eventually adjusted to a target range. Altogether, DOAC should be strongly recommended for Asian patients who require anticoagulation to reap added benefits of reduced dementia risk.

There was considerable variation among included studies in the predominant type of dementia observed (eg, Alzheimer’s was in Lee et al^26^ and vascular dementia in Mongkhon et al).^27^ HRs stratified by dementia type were not provided in most included studies, precluding a stratified meta-analysis. Although vascular dementia is the most correlated with microembolization, other types of dementia, including Alzheimer’s, have also been shown to increase in AF.^5^^,^^34^ Nevertheless, given the steep increase in incidence rates of all types of dementia beginning at approximately 70-75 years,^48^^,^^49^ it may be possible that the AF-independent increase in dementia diluted the influence of anticoagulation in the elderly population. This could explain why the correlation between age and HR for dementia risk between DOAC and warfarin had a high heterogeneity (*I*^2^ = 70%) despite being statistically significant. To separate AF-dependent and AF-independent contributory factors to dementia, 3-arm studies (with DOAC, warfarin, and untreated groups) are needed to analyze these age-related effects. Apart from demonstrating significant associations, it is difficult to infer cause-and-effect between AF and dementia because both are end results of pathologic processes that develop over many years. Hence, it is difficult to conclude whether the effects of DOAC or warfarin treatment on dementia risk are due to their influence on AF or other background processes that also influence AF.

Despite the inclusion of different DOAC therapies in varying proportions, NMA of 3 studies showed no significant differences in dementia risk between the 4 therapies. Edoxaban, which was investigated in only 1 study, had a slight but nonsignificant edge over the other DOACs. Further head-to-head trials of various DOACs are needed to support these suggestions.

### Study limitations

The nonrandomized nature of most studies precludes a definite conclusion on the true effect of DOACs vs warfarin in lowering dementia risk. The use of disease codes for dementia may not truly encompass the entirety of cases because this only captures patients who were admitted to the hospital during the follow-up period, but not those who developed dementia but were not hospitalized and remained undiagnosed in the community. Nonetheless, the corroboration of the PSM-only subgroup analysis with the main analysis is a strong point in favor of true significant effect. PSMs reduce the effect of confounding variables and have been shown to be empirically equivalent to RCTs in generating unbiased estimates of treatment efficacy.^50^ Hence, future RCTs are needed; indeed, several are currently underway (eg, GIRAF [Cognitive Impairment Related to Atrial Fibrillation Prevention Trial; NCT01994265], BRAIN-AF [Blinded Randomized Trial of Anticoagulation to Prevent Ischemic Stroke and Neurocognitive Impairment in AF; NCT02387229], and CAF [Impact of Anticoagulation Therapy on the Cognitive Decline and Dementia in Patients With Non-Valvular Atrial Fibrillation; NCT03061006]). The incorporation of standardized tests, such as the Montreal Cognitive Assessment and Mini Mental State Exam, in these trials will allow detailed evaluation of cognitive function. Longer follow-up is also suggested; included studies had a follow-up of <5 years, despite a longer duration required for development of dementia suggested by other studies.^51^ NMA accuracy is dependent on assumptions of methodologic equivalence and similarity of patient profiles.^52^ Although methodology was grossly similar in terms of study inclusion criteria and follow-up duration, patient profiles varied considerably. The effect of rhythm control of AF on dementia risk was not analyzed due to the heterogeneity contributed by its procedural nature, although significant benefit has previously been shown.^53^

Nonetheless, the impact on dementia is likely to be multifactorial, beyond anticoagulation (or DOACs) alone, hence the move toward a more holistic or integrated care approach to AF management.^54^ Indeed, there is increasing literature on lifestyle factors and risk factor management impacting on incident dementia in patients with AF,^55^^,^^56^ which is not accounted for in this analysis. An integrated care approach to AF management has been associated with improved clinical outcomes^57^ and is now recommended in international guidelines.^58^ Of note, adherence to such an integrated care approach is associated with a lower risk of incident Alzheimer’s and vascular dementia.^59^

## Conclusions

The use of DOACs in patients with AF significantly reduces dementia risk compared with warfarin, especially among Asian patients. Nonetheless, a suggestion of reversal of this effect with increasing age merits further research in the form of randomized trials with long-term follow-up.

Perspectives **COMPETENCY IN MEDICAL KNOWLEDGE:** This meta-analysis of 10 large observational studies and 342,624 patients found that the use of DOACs in AF appears to significantly reduce dementia risk compared with warfarin, especially in Asian populations. However, the benefit appears to diminish with increasing age.

**TRANSLATIONAL OUTLOOK:** This study demonstrates yet another clinical standpoint from which DOACs show benefit over warfarin, and strengthens the existing evidence base for the use of DOACs for AF.

## Funding Support and Author Disclosures

Dr Lip served as a consultant and speaker for Bristol Myers Squibb/Pfizer, Boehringer Ingelheim, Daiichi Sankyo, and Anthem; and is co-principal investigator of the AFFIRMO project on multimorbidity in AF, which has received funding from the European Union’s Horizon 2020 research and innovation programme under grant agreement no. 899871. All other authors have reported that they have no relationships relevant to the contents of this paper to disclose.
