# Supplementary material for: Dementia Risk of Direct Oral Anticoagulants Versus Warfarin for Atrial Fibrillation: Systematic Review and Meta-Analysis
Source: JACC Asia. 2023 Sep 5;3(5):776–86. doi: 10.1016/j.jacasi.2023.07.012 (PMC10715893; doi:10.1016/j.jacasi.2023.07.012)
Supplement: Supplemental Material [file mmc1.docx]

## Supplemental Table 1. Full search strategy for the respective databases.

| **PubMed** | 273 articles |
| --- | --- |
| (atrial fibrillation[MeSH Terms] OR afib) AND (DOAC OR NOAC OR OAC OR oral anticoagulation OR warfarin OR VKA OR vitamin K antagonist) AND (dementia OR cognitive impairment OR Alzheime*) NOT ((animals [mh] NOT humans [mh])) NOT (systematic[sb] OR Editorial[pt] OR Meta-Analysis[pt]) | |
| **EMBASE** | 250 articles |
| 'atrial fibrillation'/exp AND (doac OR noac OR oac OR 'oral anticoagulation' OR warfarin OR vka OR 'vitamin k antagonist') AND ((dementia OR cognitive) AND impairment OR alzheimer) NOT ([editorial]/lim OR [erratum]/lim OR [letter]/lim OR [review]/lim) | |
| **Scopus** | 448 articles |
| ALL((atrial AND fibrillation OR afib) AND (doac OR noac OR oac OR oral AND anticoagulation OR warfarin OR vka OR vitamin AND k AND antagonist) AND (dementia OR cognitive AND impairment OR alzheimer)) AND NOT ( SRCTYPE ( b ) OR SRCTYPE ( k ) OR SRCTYPE ( p ) OR SRCTYPE ( d ) OR DOCTYPE ( bk ) OR DOCTYPE ( ch ) OR DOCTYPE ( bz ) OR DOCTYPE ( ed ) OR DOCTYPE ( er ) OR DOCTYPE ( le ) OR DOCTYPE ( no ) OR DOCTYPE ( re ) OR DOCTYPE ( sh ) ) | |

Date searched: June 23, 2022

## Supplemental Table 2. Newcastle-Ottawa Scale for cohort studies.

| Study | Selection | Comparability | Outcome | Total score | Risk of bias |
| --- | --- | --- | --- | --- | --- |
| Bezabhe 2022 | ******* | ****** | ******* | 8 | Low |
| Cadogan 2021 | ******* | ***** | ******* | 7 | Low |
| Chen 2018 | ******* | ****** | ******* | 8 | Low |
| Friberg 2017 | ******* | ****** | ****** | 7 | Low |
| Hsu 2021 | ******* | ****** | ******* | 8 | Low |
| Jacobs 2016 | ******* | ****** | ******* | 8 | Low |
| Kim 2021 | ******* | ****** | ******* | 8 | Low |
| Lee 2021 | ******* | ***** | ******* | 7 | Low |
| Mongkhon 2020 | ******* | ***** | ******* | 7 | Low |
| Sogaard 2019 | ******* | ***** | ******* | 7 | Low |

## Supplemental Table 3. GRADE (Grading of Recommendations Assessment, Development and Evaluation) table summary.

| Outcome | Relative effect (95% CI) | Number of participants (studies) | Certainty of evidence (GRADE) |
| --- | --- | --- | --- |
| *Two-arm meta-analysis* | | | |
| DOAC versus warfarin | HR 0.88 (0.80-0.98) | 337,370 (9 cohort studies) | ⊕⊕⊕⊝ Moderate ^a^ |
| DOAC versus warfarin | IRR 0.87 (0.76-1.00) | 327,926 (9 cohort studies) | ⊕⊕⊕⊝ Moderate ^a^ |
| *Network meta-analysis* | | | |
| Edoxaban versus warfarin | HR 0.830 (0.665-1.036) | 541,724 (3 cohort studies with overlap of propensity score-matched patients in several arms) | ⊕⊕⊕⊝ Moderate ^a^ |
| Rivaroxaban versus warfarin | HR 0.854 (0.763-0.955) |  | ⊕⊕⊕⊝ Moderate ^a^ |
| Dabigatran versus warfarin | HR 0.871 (0.770-0.987) |  | ⊕⊕⊕⊝ Moderate ^a^ |
| Apixaban versus warfarin | HR 0.881 (0.778-0.997) |  | ⊕⊕⊕⊝ Moderate ^a^ |
| Edoxaban versus rivaroxban | HR 0.972 (0.758-1.247) |  | ⊕⊕⊕⊝ Moderate ^a^ |
| Edoxaban versus dabigatran | HR 0.953 (0.739-1.228) |  | ⊕⊕⊕⊝ Moderate ^a^ |
| Edoxaban versus apixaban | HR 0.942 (0.731-1.215) |  | ⊕⊕⊕⊝ Moderate ^a^ |
| Rivaroxaban versus dabigatran | HR 0.980 (0.840-1.143) |  | ⊕⊕⊕⊝ Moderate ^a^ |
| Rivaroxaban versus apixaban | HR 0.969 (0.836-1.123) |  | ⊕⊕⊕⊝ Moderate ^a^ |
| Dabigatran versus apixaban | HR 0.989 (0.839-1.167) |  | ⊕⊕⊕⊝ Moderate ^a^ |

^a^ Downgraded one level due to use of evidence from nonrandomized studies only.

HR, hazard ratio; IRR, incidence rate ratio.

*GRADE Working Group grades of evidence:*

High certainty: we are very confident that the true effect lies close to that of the estimate of the effect.

Moderate certainty: we are moderately confident in the effect estimate: the true effect is likely to be close to the estimate of the effect, but there is a possibility that it is

substantially different.

Low certainty: our confidence in the effect estimate is limited: the true effect may be substantially different from the estimate of the effect.

Very low certainty: we have very little confidence in the effect estimate: the true effect is likely to be substantially different from the estimate of effect.

## Supplemental Figure 1. Subgroup analysis of hazard ratio for dementia development, restricted to studies reporting outcomes for patients aged 65-75 years.


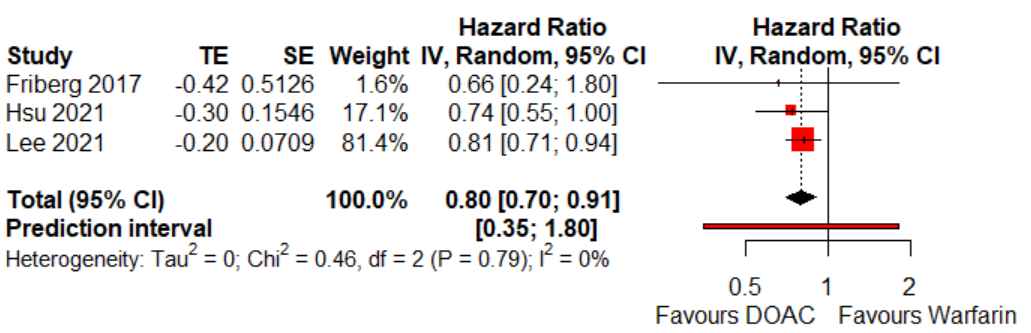


## Supplemental Figure 2. Subgroup analysis of hazard ratio for dementia development, restricted to studies reporting outcomes for patients aged ≥75 years.


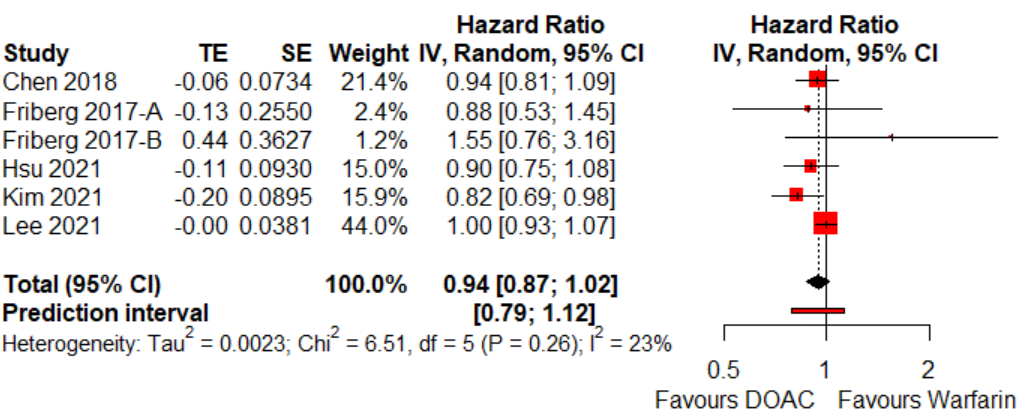


95% CI, 95% confidence interval; IV, inverse-variance; SE, standard error of treatment effect; TE; treatment effect.

## Supplemental Figure 3. Random-effects meta-analysis of incidence rate ratio for dementia development.


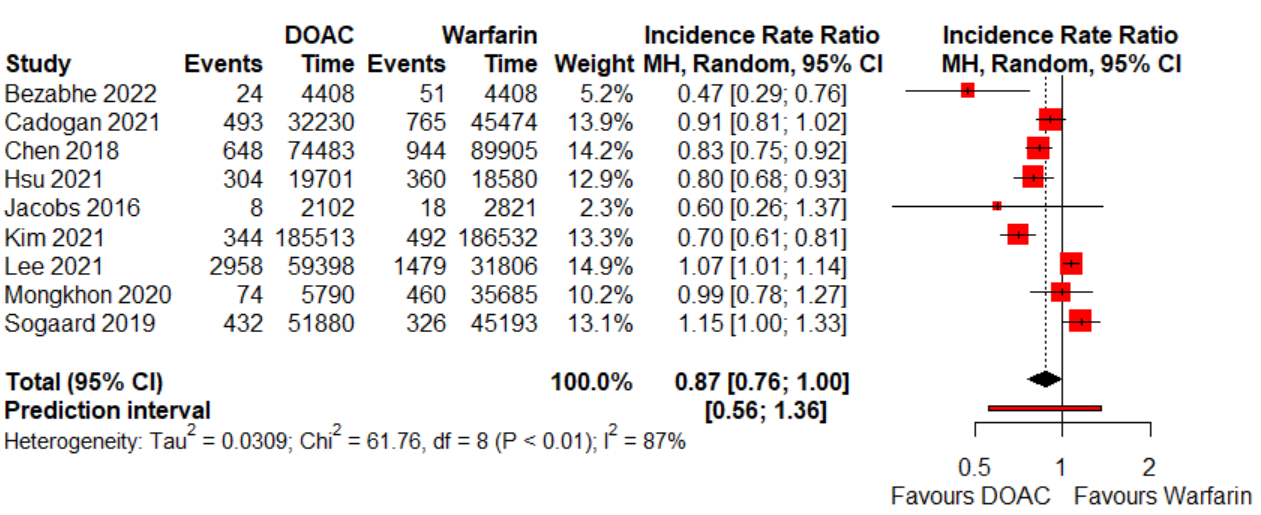


95% CI, 95% confidence interval; DOAC, direct-acting oral anticoagulant; MH, Mantel-Haenszel.

## Supplemental Figure 4. Funnel plot for dementia hazard ratio in the entire cohort.

##


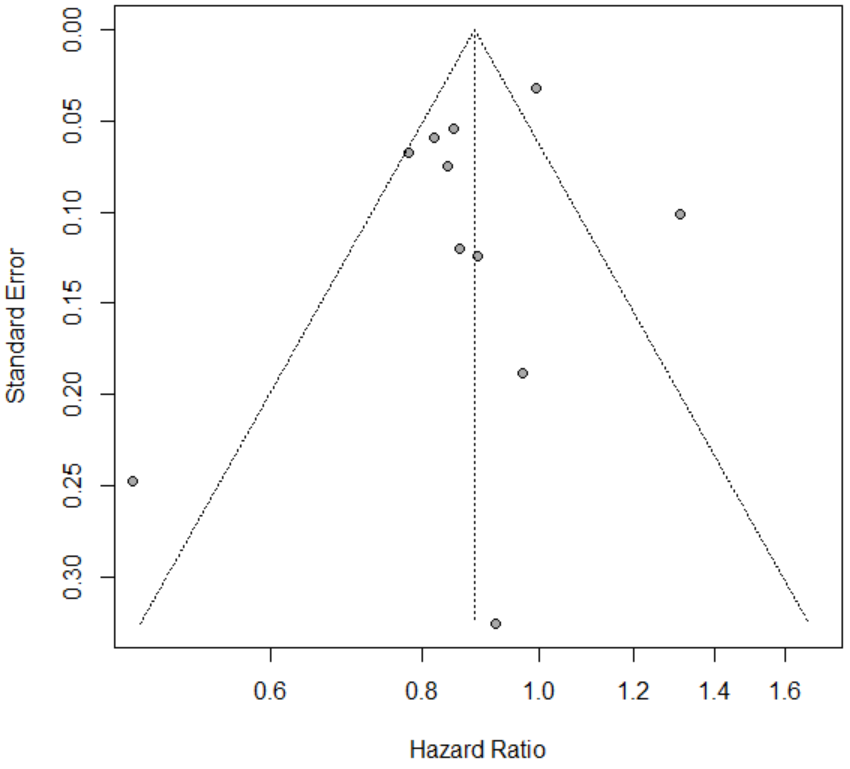


## Supplemental Figure 5. Funnel plot for dementia hazard ratio among propensity score-matched studies.


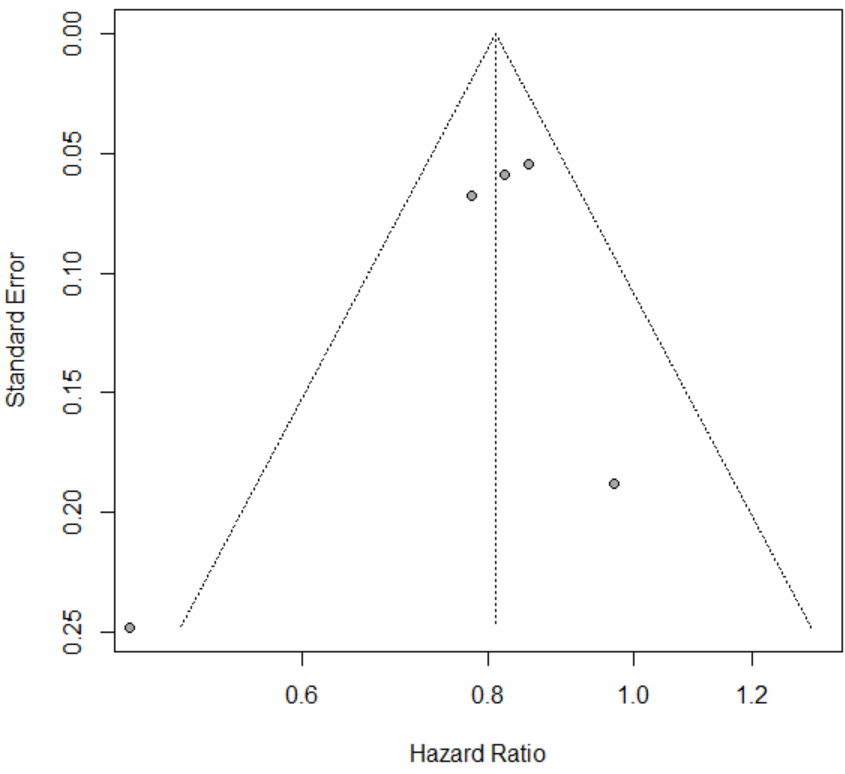


## Supplemental Figure 6. Funnel plot for dementia hazard ratio in patients aged 65-75 years.


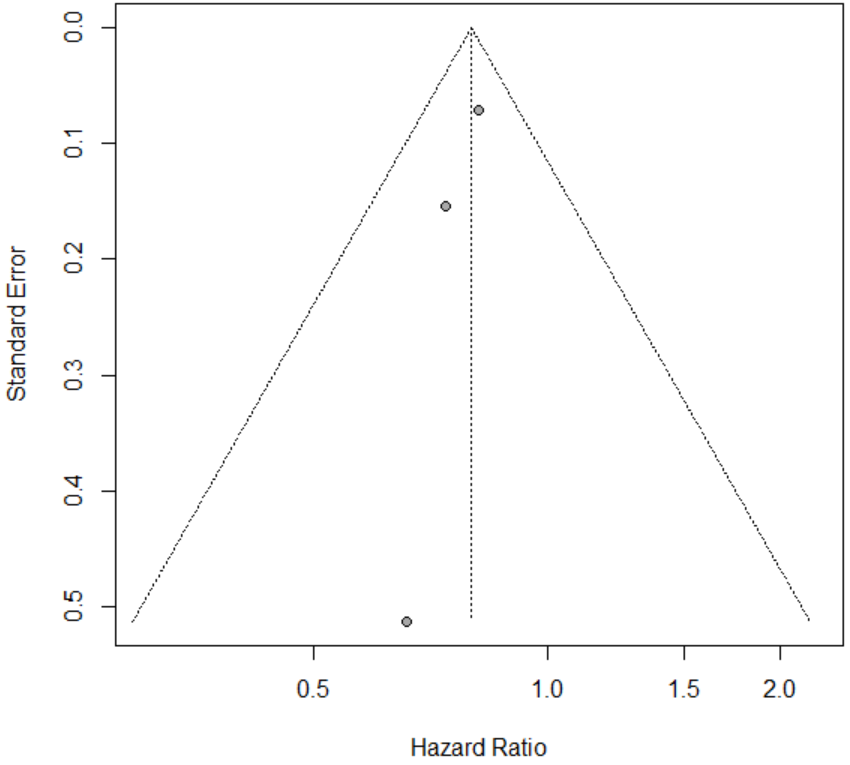


## Supplemental Figure 7. Funnel plot for dementia hazard ratio in patients aged ≥75 years.


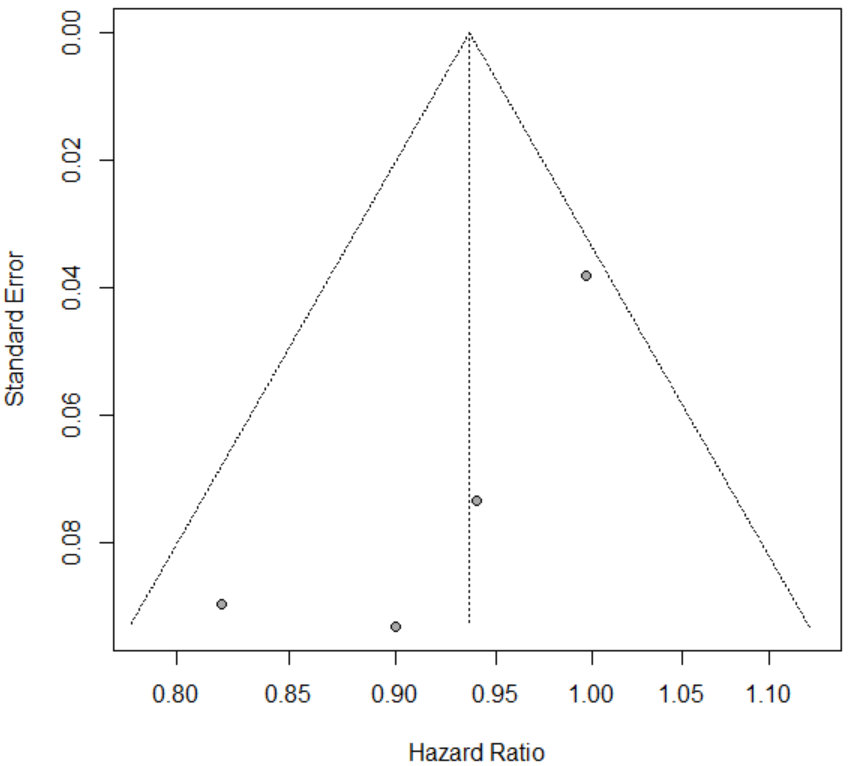


## Supplemental Figure 8. Funnel plot for dementia incidence rate ratio.


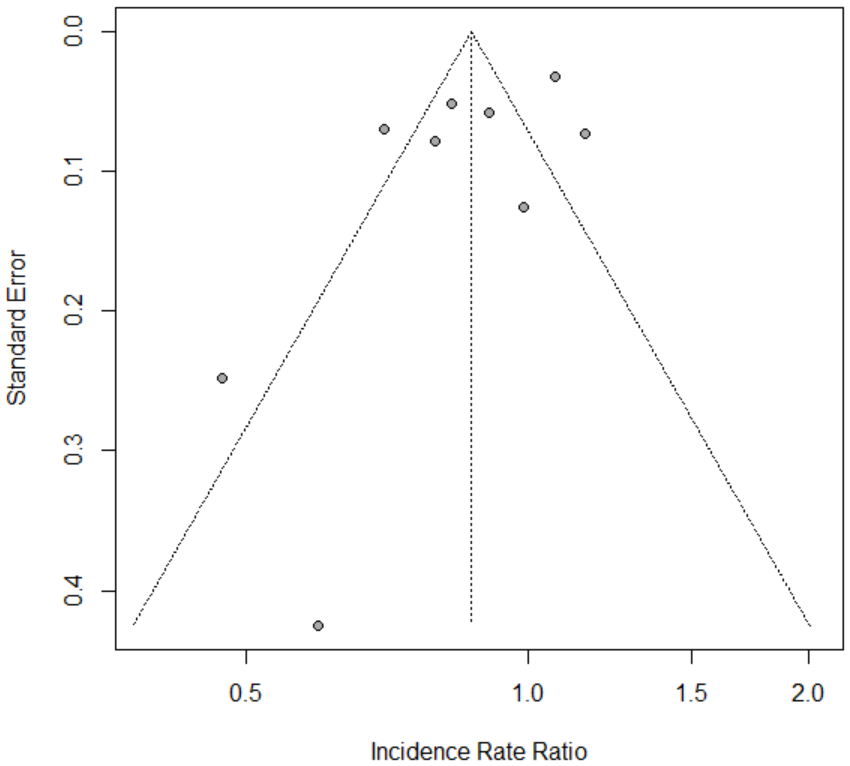


## Supplemental Figure 9. Bubble plot for age versus log (HR) for dementia.


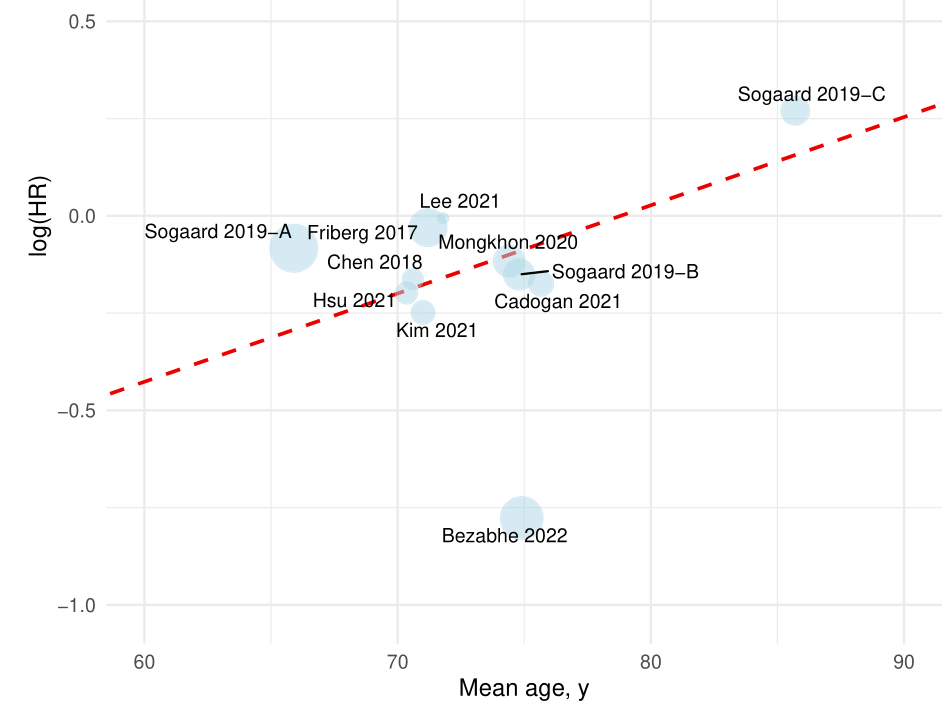


HR, hazard ratio

## Supplemental Figure 10. Network plot for network meta-analysis of dementia risk.


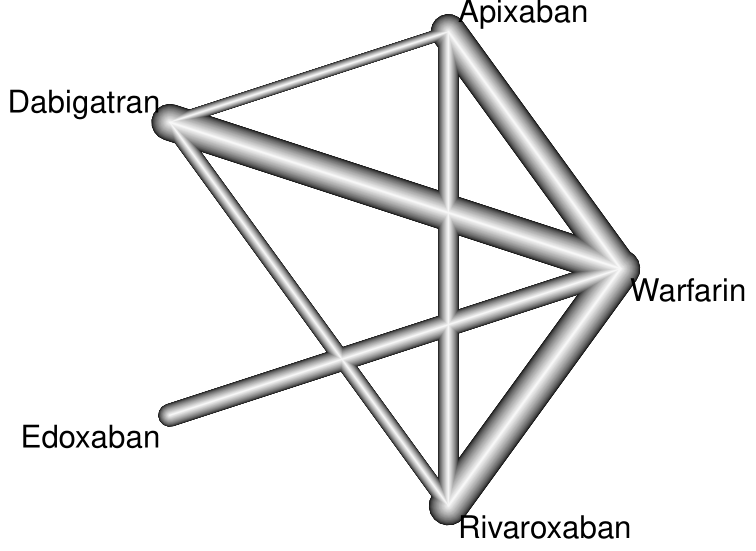


Treatment arms are represented by vertices. Edges connect treatment arms which have direct comparisons in included studies. A thicker edge corresponds to higher strength of evidence for the comparison between the two treatment vertices at its ends.

## R code

library(boot)

library(dplyr)

library(ggpubr)

library(tidyverse)

library(table1)

library(tableone)

library(ggplot2)

library(tidyverse)

library(gridExtra)

library(summarytools)

library(meta)

library(knitr)

library(metafor)

library(rms)

library(tidyr)

library(flexsurvcure)

library(grid)

library(netmeta)

library(readxl)

# meta-analysis of HR

#noac <- read_excel(paste directory) %>% as.data.frame()

dementia_meta <- metagen(TE,

seTE,

data = noac,

studlab = study,

fixed = F,

random = T,

subgroup=Region,

method.tau = "DL",

hakn = F,

prediction = F,

sm = "HR")

dementia_meta

pdf(paste(Sys.Date(),"2stage.pdf"), height=6, width=11)

tiff("Figure 2.tiff", units="in", height=7, width=8, res=300)

meta::forest(dementia_meta)

dev.off()

funnel.meta(dementia_meta)

dementia_meta2 <- metagen(TE,

seTE,

data = noac,

studlab = study,

fixed = F,

random = T,

method.tau = "DL",

hakn = F,

prediction = F,

subgroup=Region2,

sm = "HR")

dementia_meta2

meta::forest(dementia_meta2)

#subgroup 75y/o

noac.75 <- noac%>%filter(hr.75>0)

noac.75

dementia.75_meta <- metagen(TE.75,

seTE.75,

data = noac.75,

studlab = study,

fixed = F,

random = T,

method.tau = "DL",

hakn = F,

prediction = F,

sm = "HR")

dementia.75_meta

dementia.75_meta %>% funnel.meta()

pdf(paste(Sys.Date(),"2stage_75_sub_2.pdf"), height=6, width=11)

meta::forest(dementia.75_meta)

dev.off()

#subgroup 65-75y/o

noac.6575 <- noac%>%filter(hr.6575>0)

noac.6575

dementia.6575_meta <- metagen(TE.6575,

seTE.6575,

data = noac.6575,

studlab = study,

fixed = F,

random = T,

method.tau = "DL",

hakn = F,

prediction = F,

sm = "HR")

dementia.6575_meta

dementia.6575_meta %>% funnel.meta()

pdf(paste(Sys.Date(),"2stage_6575_sub_2.pdf"), height=6, width=11)

meta::forest(dementia.6575_meta)

dev.off()

noac.psm <- noac%>%filter(type=="PSM")

noac.psm

dementia.psm_meta <- metagen(TE,

seTE,

data = noac.psm,

studlab = study,

fixed = F,

random = T,

method.tau = "DL",

hakn = F,

prediction = F,

sm = "HR")

dementia.psm_meta

dementia.psm_meta %>% funnel.meta()

pdf(paste(Sys.Date(),"2stage_psm_sub.pdf"), height=6, width=11)

tiff("Figure 3.tiff", units="in", height=4, width=8, res=300)

meta::forest(dementia.psm_meta)

dev.off()

#meta-regression template

out.reg <- noac

m.gen.reg <- metareg(m.gen, ~variables)

summary(m.gen.reg)

out.reg.table

##bubble plot template

data %>%

arrange(desc(pop)) %>%

mutate(country = factor(country, country)) %>%

ggplot(aes(x=gdpPercap, y=lifeExp, size=pop, color=continent)) +

geom_point(alpha=0.5) +

scale_size(range = c(.1, 24), name="Population (M)")

##metainc

dementia_ir <- metainc(event.e=n_dementia_doac,

time.e=personyear_doac,

event.c=n_dementia_warfarin,

time.c=personyear_warfarin,

data = noac,

studlab = study,

fixed = F,

random = T,

method.tau = "DL",

hakn = F,

prediction = F,

sm = "IRR")

dementia_ir

dementia_ir %>% funnel.meta()

pdf(paste(Sys.Date(),"2stage_IRR.pdf"), height=6, width=11)

meta::forest(dementia_ir)

dev.off()

#NMA

out.os

os.net <- netmeta(TE = TE,

seTE = seTE,

treat1 = arm1,

treat2 = arm2,

studlab = Ref,

data = out.os,

sm = "HR",

fixed = F,

random = T,

reference.group = "Warfarin",

details.chkmultiarm = TRUE,

sep.trts = " vs ")

os.net

decomp.design(os.net)

pdf(paste(Sys.Date(),"network_graph.pdf"), height=5, width=7)

netgraph(os.net)

dev.off()

os.league <- netleague(os.net)

os.league

os.p <- netrank(os.net, small.values = "good")

os.p

os.p1 <- as.data.frame(os.p$Pscore.random)

pdf(paste(Sys.Date(),"network_forest.pdf"), height=5, width=7)

tiff("Figure 4.tiff", units="in", height=4, width=8, res=300)

forest(os.net)

dev.off()
